# Diagnostic Validity of Digital Imaging Fiber-Optic Transillumination (DIFOTI) and Near-Infrared Light Transillumination (NILT) for Caries in Dentine

**DOI:** 10.3390/jcm9020420

**Published:** 2020-02-04

**Authors:** Ana Marmaneu-Menero, José Enrique Iranzo-Cortés, Teresa Almerich-Torres, José Carmelo Ortolá-Síscar, José María Montiel-Company, José Manuel Almerich-Silla

**Affiliations:** Stomatology Department, University of Valencia, 46010 Valencia, Spain; menero@alumni.uv.es (A.M.-M.); j.enrique.iranzo@uv.es (J.E.I.-C.); Teresa.almerich@uv.es (T.A.-T.); jose.c.ortola@uv.es (J.C.O.-S.); jose.m.almerich@uv.es (J.M.A.-S.)

**Keywords:** cariology research, diagnostic Systems, radiology, imaging, cavities

## Abstract

The objective of the study is to analyse the available evidence for the validity of the transillumination method in the diagnosis of interproximal caries. Bibliographic searches were carried out in three data bases (PubMed, Embase, Scopus) with the key words “Transillumination AND caries”. A total of 11 studies were selected for the qualitative analysis and meta-analysis. In the qualitative analysis, both in vivo and in vitro studies were included. The gold standards were tomography, digital radiography, and clinical visual diagnosis. The meta-analysis determined the sensitivity, specificity, and area below the ROC curve relative to the transillumination method in the diagnosis of caries in dentine. Meta-analysis results obtained for transillumination gave a sensitivity value of 0.69 (confidence interval: 0.54–0.81), a specificity value of 0.89 (confidence interval: 0.61–0.98), while giving an AUC value of 0.79 (confidence interval: 0.67–0.87). Transillumination is a method offering moderate validity in the diagnosis of carious lesions in dentine, there is no strong evidence that may enable us to affirm that transillumination may fully substitute X-rays in the complementary diagnosis of carious lesions

## 1. Introduction

Dental caries is an infectious disease stemming from the interaction of various factors. It amounts to a multifactorial disease because, for the pathogenic process to start, the presence of microorganisms that interact with cariogenic foodstuffs in the diet of a likely patient is required. Consequently, the pathology unfolds within its evolution period. The resulting situation involves the characteristic progressive destruction of calcified tissue in teeth due to the pathogenic action of acidic products arising in the bacterial metabolism of fermented carbohydrates in the patient’s diet [1,2].

It is a disease with a diminished prevalence in the last two decades, particularly in developed countries, due to the widespread introduction of fluoride in toothpaste [1]. Notwithstanding this, it is a common disease that negatively impinges upon quality of life in a large part of the world’s population and continues to be one of the main causes of tooth loss. It is therefore considered to be a public health problem warranting a correct diagnosis, above all in terms of attaining a reliable diagnosis in initial pathological phases.

Currently, minimally invasive dentistry calls for the development of a diagnostic technique for carious lesions in the earliest stages of the disease and hence the sensitivity as well as the specificity of these new methods needs to be established [2].

The methods that have been developed over the last decade are quite varied in terms of their mechanism, but they all have in common that there is a resolve to substitute the classical X-ray diagnosis according to the bite-wing method [2]. In this study, we focus on transillumination methods: Digital Fiber Optic Transillumination (DIFOTI), and Near-Infra-red Light Transillumination (NILT).

Transillumination is a method based on optic fibre technology whereby a tooth is targeted by high-intensity white light emanating from a hand-held device [3]. Tissue with caries, being more porous than healthy tissue, absorbs far more light enabling us to observe that the surrounding tissue is whiter and more opaque, whereas the lesion appears darker because carious lesions disperse visible light [3,4]. This method may be used on any dental surface of the patient, particularly in interproximal lesions of anterior teeth, as in this case, the bucco-lingual enamel thickness is lower than in posterior teeth [3]. The light is propagated towards the tooth by way of optic fibres and has the sufficient intensity to pass through the dental structure to reach areas difficult to view with the naked eye [4].

Eventually, this method was modified by adding a digital camera, called a Digital Imaging Fiber-Optic Transillumination (DIFOTI). This instrument enables in vivo digital images to be captured with only very scant distortion when the light transmitted passes through the tooth and becomes a detectable signal read by a computer; it is then instantaneously displayed on a screen [5]. To this end, DIFOTI has been designed with a handpiece, a control panel, specific software, a personalised image-recording system, a pedal, as well as different projection possibilities according to the surface to be examined (one for occlusal surfaces and the other for interproximal surfaces).

Over the past two decades, interest in transillumination as a method for diagnosing carious lesions led to the firm Kavo^®^ presenting in 2012 the product known as DiagnoCam^®^ as a further development of DIFOTI [6]. This is a device based on transillumination whereby invisible near-infrared light oscillating between 700 nm and 1500 nm NILT (Near-Infrared Light Transillumination), this being the main difference with respect to FOTI and DIFOTI [7].

It was believed that this variation in longitude of wavelength of light would have a promising effect in terms of the detection of early-stage carious lesions, as it had been shown that light with the said characteristics disperses itself less and hence is able to penetrate more deeply in dental tissue, thereby enabling a better transmission of light, offering hence a considerable benefit [6,7,8]. This method entails, according to the absorption and dispersion of light, a good contrast between carious areas and those healthy areas surrounding the lesion, facilitating detection.

In accordance with the principle of “as low as reasonably achievable”, which involves keeping to a minimum the amount of radiation that patients can be exposed to, the X-rays are only indicated when this procedure is necessary, particularly when the patients are children. This, in consideration of the reduction in the prevalence and slower progress of carious lesions, due to the use of fluorides, has resulted in radiology techniques attracting some controversy. New diagnostic methods exist as alternatives to ionizing radiation, but all require prior studies to affirm that their clinical application offers a greater detection of interproximal caries. 

For the reasons outlined above, the objective of our study was to analyse the available evidence for validating the transillumination method in the diagnosis of interproximal caries at the dentine level.

## 2. Experimental Section

To meet the objective, we posed the following PICO question: Does transillumination (I) give good sensitivity and specifity values (O) in the general population seen in daily practice (P) compared to X-rays (C)?

### 2.1. Article Search and Selection Strategies

The search was carried out in 3 databases: PubMed, Scopus and Embase, applying the following search equations: 

For PubMed the equation was: “((“transillumination”(MeSH Terms) OR “transillumination”(All Fields)) OR DIFOTI (All Fields) OR NILT (All Fields)) AND (“dental caries” (MeSH Terms) OR (“dental” (All Fields) AND “caries” (All Fields)) OR “dental caries” (All Fields)) AND (“1997/01/01”(PDAT): “2020/12/31”(PDAT))”, obtaining 165 articles. 

For Scopus, the equation was: “(TITLE-ABS-KEY ((transillumination OR difoti OR nilt)) AND TITLE-ABS-KEY (“dental caries”) AND PUBYEAR > 1996”, obtaining 175 articles.

For Embase, the equation was: “(transillumination OR difoti OR nilt) AND ‘dental caries’ AND (1997-2020)/py”, obtaining 117 articles.

The search took place on 28 January 2020. Years before 1997 were excluded from the search as the first article found about DIFOTI or NILT was dated in that year. Duplicates were searched and excluded using RefWorks^®^ tool. Any article was found through revision of the references of the selected articles.

Two qualified reviewers (AM-M and JMM-C), independently selected the articles. Whenever there was disagreement, a third reviewer (JEI-C) decided whether to include an article in the study. A Kappa value of 0.91 was obtained to determine inter-reviewer reliability. The initial selection was carried out by reading article titles and abstracts. Whenever the information was found to be insufficient, the whole article was read in-depth before a final decision was taken.

Several requisites were established for including the studies in the qualitative synthesis: the articles included in the study had to have been available in English and included the key words in the title or abstract. Furthermore, a representative sample of any age was required in relation to transillumination use, either with visible or invisible light.

Further still, articles not containing statistical data or those that contained key words though not related to the study topic, related to temporary teeth or that used transillumination for the diagnosis of occlusal legions were excluded. 

### 2.2. Article Quality Analysis 

Article quality was established by QUADAS-2 [9] which evaluates quality levels in diagnostic precision studies according to flow and timing, reference standards, index test and patient selection. 

### 2.3. Data Extraction

To determine the diagnostic precision of dental caries in the index test in terms of agreement with the benchmark test or gold standard, data mining took place for the values of sensitivity, specificity and area under the ROC curve, provided that stage D3 of caries had been demonstrated (caries that have reached the dentine).

### 2.4. Statistical Analysis

For the quantitative analysis, a meta-analysis was carried out based on data obtained in articles included in the systematic literature review, hence allowing values for sensitivity, specificity and area under the curve (ROC) to be obtained for transillumination methods used in the diagnosis of dental caries. 

The combinations of the studies were carried out according to the random effects model and assuming intra- and inter-study variability. The calculation of statistical heterogeneity between the studies was done in accordance with Cochran’s Q-test and p-value, as well as in I^2^. A sub-group analysis was carried out according to whether the study had been carried out in vivo or in-vitro as well as a meta-regression through the Maximum Likelihood Method.

A sensitivity analysis was carried out in the studies included in the meta-analysis by using the one study removed method, thereby establishing in which way the exclusion of each of the studies may influence the global estimation. 

Lastly, to analyse the publication bias, the funnel plot was obtained for sensitivity, specificity, and AUC values by analysing the symmetry of the studies. Furthermore, by making use of the Duval and Tweedie’s Trim and Fill Method, one or various studies were engaged to establish how the effect size might condition the global estimation. Egger’s intercept was also applied with two-tailed *p*-values. The statistics analysis was performed using Comprehensive Meta-analysis v.3 and the significance level was set at *p* < 0.05.

## 3. Results

### 3.1. Bibliographic Search

**Figure 1:**
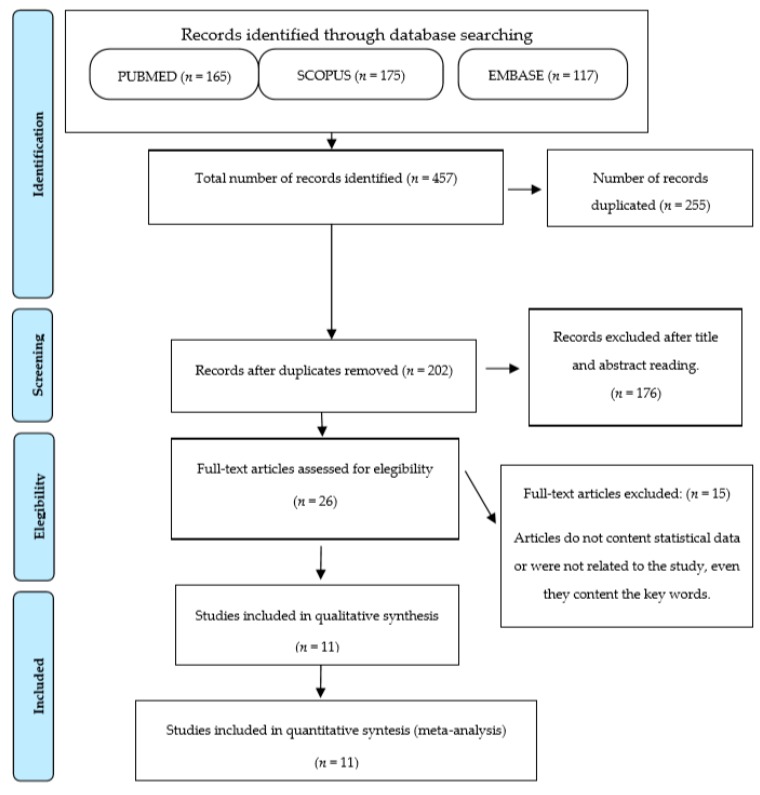
Flow chart.

A total 457 articles were obtained after applying the search criteria (165 in PubMed, 175 in Scopus y 117 in Embase). Once repeated articles were eliminated, the number of articles was reduced to 202; of these a further selection was carried out after reading their abstracts in order to fine-tune the search and 26 articles were finally selected. The whole text of each article was then read to finalise the bibliographic search. A total of 12 articles met the abovementioned inclusion and exclusion criteria. The following flow chart represents selection process (Figure 1) according to the PRISMA criteria [10].

### 3.2. Qualitative Analysis

A total of 11 articles obtained in the bibliographic review were studied (Table 1); 7 were in vitro studies (VT) of previously extracted teeth with caries [11,12,13,14,15,16,17] and which has had been treated in order to simulate the oral cavity conditions in the study by placing them in supports that imitated soft tissue to obtain more realistic results. The remaining 4 articles were carried out in vivo (VV) [6,7,18,19] in a specific sample (*n*).

The methodology used in most of the studies included in the meta-analysis involved carrying out a bite-wing X-ray in each tooth with caries; this tooth could be an extracted one or otherwise, it was still present in the mouth. The examiners gave a value that corresponded to the extent of the carious lesion and, subsequently, the same tooth was observed by each examiner using a transillumination method and then determined the extent of the caries. The examiners had been previously given training in transillumination instrument use. The same examination was repeated one week later and once again, the dental lesion was evaluated, with the exception of the study by Maia et al. [14] where the repetition took place at 6 months. 

At a later stage, the severity of each of carious lesions was verified through X-rays and transillumination; clinical confirmation of this appears in 3 articles [6,7,18], in other words, an aperture of the cavity takes place to see the severity of the carious lesion. One of these uses a CAT scan [11], while the remaining 4 studies [12,13,14,19], established the histology as the gold standard; here tooth sections were observed with an optical microscope.

The number of examiners ranged between 2–3 except in the article by Ástvaldsdóttir et al. [12], where the lesions were diagnosed by 8 examiners and a sole mean value was calculated for each method. It should be pointed out that all of the examiners were dentists except in the study by Baltacioglu I. et al. [6], where one examiner was a radiologist. 

The said articles have studied the values for sensitivity (Sn), specificity (Sp) and AUC in transillumination (DIFOTI or NILT) and the performance of the digital X-ray with reference to a gold standard; the data generated are set forth in Table 1.

**Figure 2:**
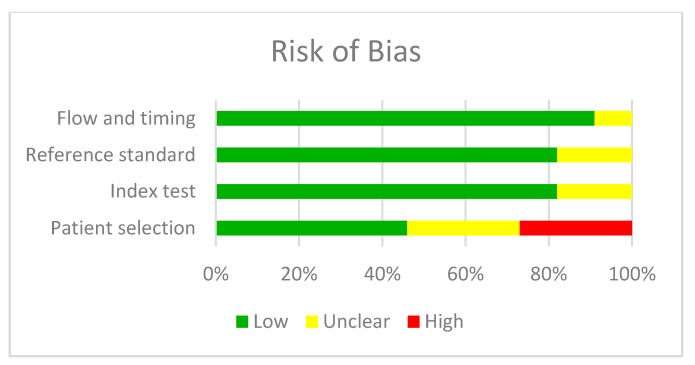
Risk of bias in the studies analysed.

**Figure 3:**
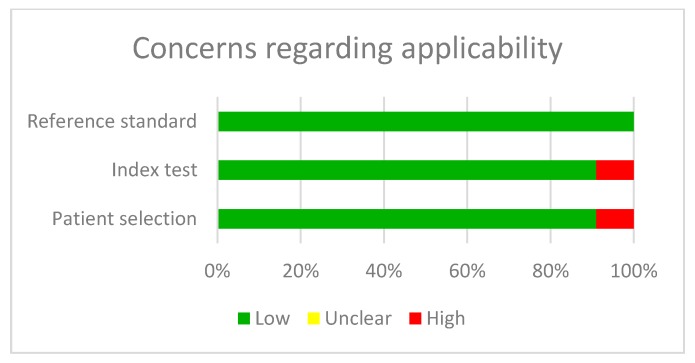
Concerns regarding applicability of the results of the studies analysed.

The quality analysis of the studies was carried out with the QUADAS-2 tool and the biggest concern was the selection bias in patients (Table 2 and Figure 2 and Figure 3).

### 3.3. Quantitative Analysis

The quantitative synthesis studies were divided into sensitivity, specificity and AUC at the D3 threshold (caries in dentine). 

### 3.4. Sensitivity

**Figure 4:**
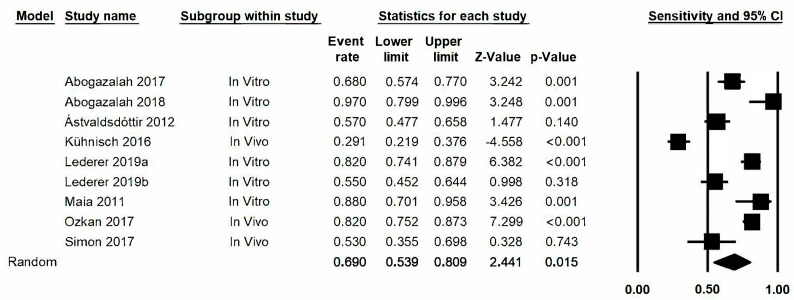
*Forest plot* for sensitivity.

The estimation of sensitivity in the transillumination method, represented by a forest plot (Figure 4) obtained a value of 0.69, 95% confidence level: 0.54–0.81. The meta-analysis presented a high degree of heterogeneity among the various studies and gave a Q-value of 113.1 (*p* < 0.05) and *I*^2^ with a value of 92.9%. 

### 3.5. Analysis Based on Subgroups and Metaregression

**Figure 5:**
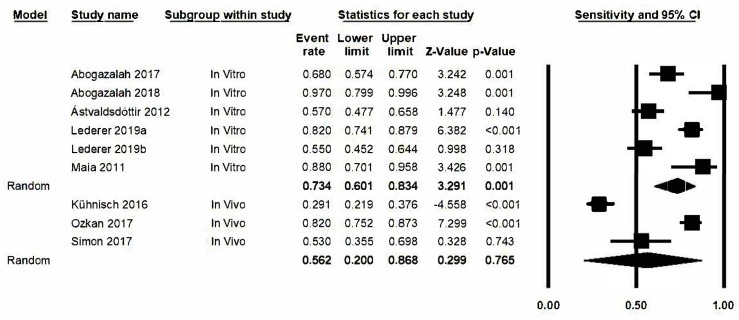
*Forest plot* for sensitivity by subgroups.

To determine if a possible source of results heterogeneity was the fact that there were teeth subject to either in vivo or in vitro conditions, an analysis based on sub-groups (in vitro/in vivo) was carried out. It was observed that heterogeneity was maintained with a Q-value of 71.3 (*p* < 0.05), an *I*^2^ value of 97.2% and a Q-value of 340000000032 There were no significant differences between the groups (Q test total between groups *p*-value = 0.39 (Figure 5). Neither did metaregression analysis detect an effect attributable to the type of study in the estimation (*p*-value: 0.189, *R*^2^ = 0.15).

### 3.6. “One Study Removed” Analysis

With the one study removed option, a sensitivity estimation was calculated for all of the studies excluding each one at a time in order to gauge how the sensitivity of the rest of the studies might be affected; using this approach, it was observed that there was no significant improvement in the global estimation by the said elimination of one of the studies (Figure S1).

### 3.7. Specificity

**Figure 6:**
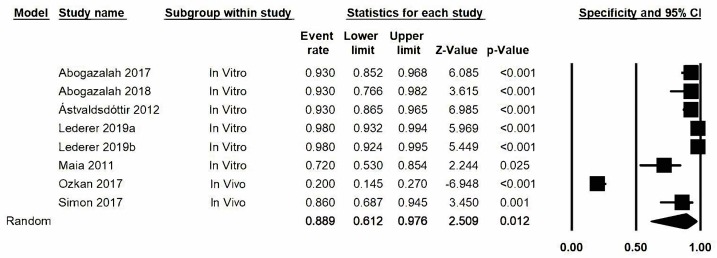
*Forest plot* for specificity.

The estimation of specificity was represented as a forest plot (Figure 6) obtaining a value of 0.89, with a lower limit confidence interval value of 0.61 and an upper limit value of 0.98. We stress here that the specificity estimation that most differed from the rest of the studies was the one by Ozkan, 2017 (0.20). The degree of heterogeneity between studies was very high and presented high levels of significance: Q-value: 210.2 (*p* < 0.05) and *I*^2^: 96.7%.

### 3.8. Analysis Based on Subgroups and Metaregression

**Figure 7:**
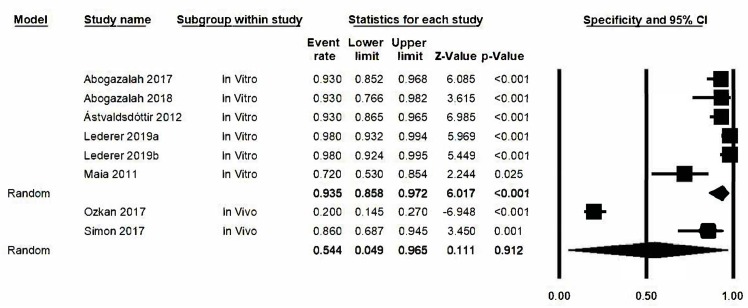
*Forest plot* for specificity by subgroups.

An estimation was obtained for the lower degree of specificity in the in vivo subgroup with the broadest confidence interval (0.049–0.965) (Figure 7). However, the degree of heterogeneity is maintained and this effectively rules out that this is the source of heterogeneity as the in vivo subgroup obtains a Q-value of 32.4 (*p* ≤ 0.05) and *I*^2^ has a value of 96.9%, while in the in vitro subgroup we find a Q-value of 22.1 (*p* = 0.001) and *I*^2^ has a value of 77.3%. There are no significant differences between the groups (Q test total between groups *p* value = 0.135). Nonetheless, the metaregression analysis detected an effect due to the type of study in the estimation (model-based test; *p*-value = 0.01, *R*^2^ = 0.52). 

### 3.9. “One Study Removed” Analysis

With the one study removed option, a calculation was made for specificity estimations for all of the studies by excluding, each study, one at a time, and hence observing that all of them presented a broad 95% confidence interval; however by excluding the study by Ozkan [7] the confidence interval obtained, in comparison with the rest, diminishes; its lower limit value is 0.86, while its upper limit value is 0.96 given that in this article the results obtained are quite different with respect to those in other studies (Figure S2).

### 3.10. Area under the Curve (AUC)

**Figure 8:**
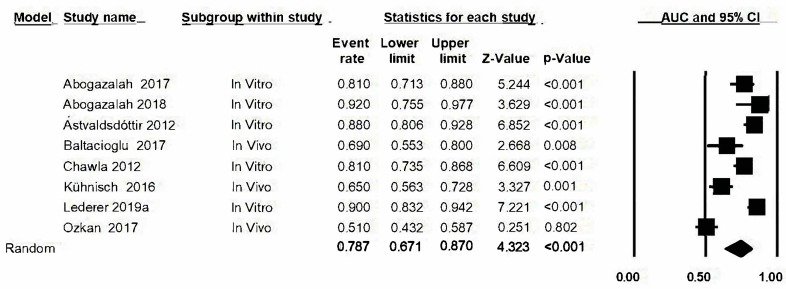
*Forest plot* for AUC.

The area under the curve (AUC) was estimated by combining the sensitivity estimation and the specificity estimation from the transillumination method. The forest plot for AUC (Figure 8) is represented and gave a value of 0.79 with a confidence interval: 0.67–0.87. The estimations for AUC are similar in all studies except in Ozkan’s case (0.51). There is heterogeneity between the studies as there is a Q-value of 78.9 (*p* ≤ 0.05) and *I*^2^: 91.1%.

### 3.11. Analysis Based on Subgroups and Metaregression

**Figure 9:**
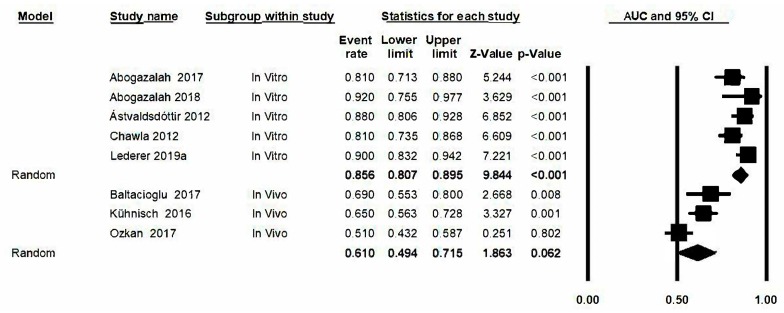
*Forest plot* for AUC by subgroups.

**Figure 10:**
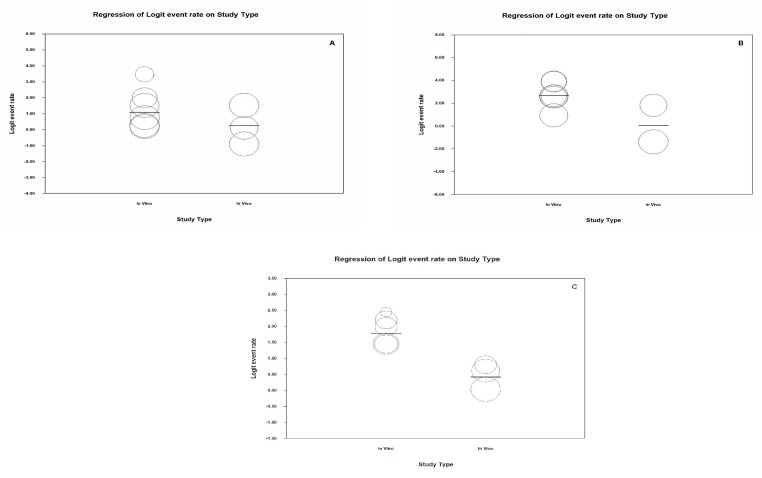
Scatter Plots meta-regression for Sensitivity (**A**), Specificity (**B**) and AUC (**C**) for subgroups (in vivo/in vitro).

Given that analysis was carried out in subgroups (in vivo/in vitro), represented hereunder in the forest plot (Figure 9), a lower AUC estimation was found in the in vivo studies (0.61, confidence interval between 0.49 and 0.72) with respect to the in vitro studies (0.86, confidence interval between 0.81 and 0.90). The Q-values and *I*^2^ values for the in vivo group were also determined: 8.1 (*p* = 0.02) and 75.3% respectively; in this subgroup however, heterogeneity for the in vitro subgroup diminishes and a significant Q-value is not obtained: Q-value: 6.9 (*p* = 0.139) and *I*^2^: 42.4. Significant differences between both groups are detected (Q test total between groups, *p* < 0.05). Metaregression analysis (Figure 10) confirms the group effect in the estimation (model-based test; *p*-value<0.05, *R*^2^ = 0.89). The studies conducted under in vivo conditions present a lower degree of diagnostic precision than in the in vitro studies.

### 3.12. Analysis “One Study Removed” 

One study removed analysis, to establish how each study affects the AUC estimation, did not reveal any study with values that differed excessively from the rest; in all of the studies, the values were similar, both in the lower limit and in the upper limit of the confidence interval (Figure S3).

## 4. Publication Bias

### 4.1. Sensitivity 

**Figure 11:**
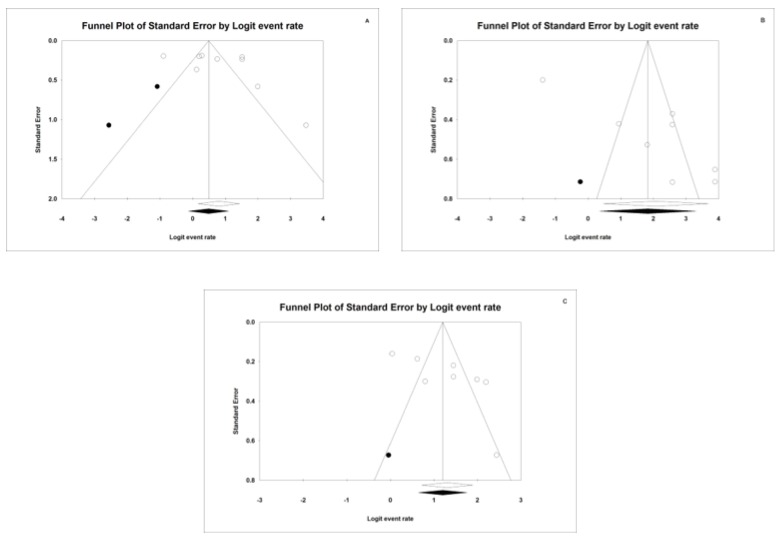
Funnel Plots for Sensitivity (**A**), Specificity (**B**) and AUC (**C**).

Publication bias analysis for sensitivity is represented by a funnel plot (Figure 11A). The Duval and Tweedie’s Trim and Fill method does not implicate any study in the analysis and we can, therefore, claim that there is no publication bias as the estimation obtained did not change. Egger’s Intercept was valued at 3.82; range: (from −4.05 to 11.69), while *p*-value = 0.289 and hence no publication bias was observed.

### 4.2. Specificity

To analyse publication bias for specificity, we obtain a funnel plot for this component (Figure 11B). It can be observed that all the studies are located at one side of the mean estimation. The estimation obtained for specificity between all of the studies analysed, i.e., white rhombus, (0.89, 95% confidence interval (0.61–0.98) significantly differs from the case of implicating new studies, i.e., black rhombus (0.86, 95% confidence interval (0.58–0.97) and hence there is a shift towards the right. Specificity is hence affected by publication bias. This can be corroborated with Egger’s Intercept which has a value of 10.6 (5.1–16.1); *p*-value = 0.01, indicating publication bias.

### 4.3. AUC

When analyzing publications bias through funnel plot, there is no publication bias for AUC, as is illustrated in Figure 11c. The representation of the estimation obtained in all the articles, i.e., white rhombus, does not differ when other articles are implicated in the Trim and Fill Method. However, this absence of bias cannot be corroborated by way of Egger’s Intercept, with a value of 7.1 (0.85–13.4); *p*-value = 0.03.

## 5. Discussion

This study has explored, both in a qualitative and quantitative manner, the results for sensitivity, specificity and area under the curve in relation to caries diagnosis in transillumination with optic fibre. Considering that the sensitivity of transillumination is its capacity to detect the presence of caries in D3 given that a value of 0.69 has been obtained (confidence interval: 0.54–0.81), mention must be made that transillumination detects two out of three lesions; it follows therefore that with this method one third of carious lesions would not be diagnosed. 

On the other hand, the ability of the transillumination technique to detect health in healthy dentine at D3 stage, in other words, the specificity this method has a value of 0.89 (confidence interval: 0.61–0.98). This value exceeds the 80% level, and hence this can be a reasonable result. In the quantitative analysis carried out for specificity, we found ourselves with one article less in the said analysis as Kühnisch [18] decided to exclude from his study cases of healthy teeth or teeth with caries in the enamel; this author does not consider its validation to be ethical and hence specificity cannot be calculated due to the absence of controls. 

By combining both values in AUC, the value continued to be below the limit considered to be good, as the result obtained was 0.79 (confidence interval: 0.67–0.87).

The interpretation of the said estimations may invite us to affirm that transillumination is not a good diagnostic method for caries if it is used in isolation; the results obtained in the quantitative analysis does not reach in some cases the required 80% threshold. The results are moderate except for specificity, in which case the performance level is surpassed. Notwithstanding this, most qualitative analysis authors affirm that transillumination would be a useful method, as it generates results that are categorised as good in around 60 to 70% of cases. An exception is Bin-Shuwais who obtains an estimation of 0.84 in this author’s in vivo study [20]. All of them consider, in view of the results of their studies, that transillumination presents results which are similar or even better in relation to sensitivity, specificity, and AUC than the case of X-rays either in vivo or in vitro even though there are certain limitations [20,21,22,23,24,25]. Therefore, in spite of the differences in the results obtained in other studies, the said qualitative review authors, together with other authors, affirm that transillumination is an alternative in the detection of carious lesions on interproximal surfaces and that it is valid and reliable when reservations exist in X-ray use; this occurs when radiation use is to be avoided or when there may be any other reason that prevents a radiological intervention.

One of the main advantages offered by X-rays is that the magnitude of may be observed in terms of its relationship with dental pulp, an aspect that is not possible with transillumination in spite of offering us a three-dimensional image of carious lesions [7,18]. Therefore, authors such as Kühnisch prefers X-rays in spite of reasonably good results obtained in transillumination even though this author does not exclude its use in other complementary diagnostic techniques to predict the severity of caries and the need or otherwise for operative treatment [18].

The main problem in our study is the high degree of heterogeneity obtained in the quantitative analysis. An attempt is made to explain this heterogeneity through subgroup analysis (in vitro/in vivo), as some authors have stated that the efficacy of these methods, applied in ideal conditions, i.e., in vitro, differ in their measurement when applied in vivo, since in soft tissue, saliva, periodontal tissue or in factors related to the patient, there may be interference in the procedure [12,24]. After carrying out analysis by subgroups, it was observed that this was not the source of the said heterogeneity as it is maintained both in the in vitro group and in the in vivo group in terms of sensitivity and specificity. However, through meta-regression, we have observed significant differences, both in specificity as well as in AUC for both in vitro and in-vivo studies, where the difference is in favour of in vitro.

Another possible source of heterogeneity could be the method used by each author to carry out the study, given that transillumination includes optical fibre and visible light wavelength (DIFOTI) as well as the non-visible kind (NILT). Under the same conditions whether in vitro or in vivo, some authors obtained different values for sensitivity and specificity. Both methods have the same operating mechanism for the diagnosis of caries but those which use an invisible light wavelength are regarded to be more promising. As various authors have pointed out, with a light emission wavelength of 13,010 nm, better images may be obtained [14,22,25,26]. If this were not the cause of the high degree of heterogeneity, we could also consider the validation or “gold standard” used in each study as histology techniques for in vitro studies; this entails an objective and very precise “gold standard”. It must be stressed that the other studies have based the “gold standard” on the clinical aperture of carious lesions, which would be affected by the subjectivity of the clinician as the threshold between healthy tissue and the presence of lesions cannot be firmly established.

Despite carrying out bibliographic searches with the same terms in three different databases, we located a small number of articles in the literature that focus on sensitivity, specificity or AUC. This creates a major limitation for our study together with the ethical problem of the clinical aperture of lesions that only affect enamel, and which may respond to remineralisation under conservative treatment; the implication here is that a “gold standard” validation of the said lesions is effectively prevented. The clinician is compelled to distinguish between two situations: the presence of lesions only in enamel (D1) and caries in enamel and dentine (D3) [6,7,18,19]. The validation of both types of lesions is only possible in in vitro studies [11,12,13,14,19] due to the previously described limit; the outcome is that quantitative analysis only considers D3 lesions due to the lack of studies that include incipient lesions which only affect the enamel.

Furthermore, the studies include various examiners and there were up to 8 examiners in the study by Astvaldsdottir et al. [12] who obtained very different values, given that in transillumination, the examiner can exert a big influence. This is why a good prior grading of examiners is necessary if good results are to be obtained [7,11,12,13,18]. This is an important question to bear in mind when studying new caries-diagnosing devices based on transillumination when we wish to compare results with digital X-rays; this is particularly pertinent when considering bite-wings since clinicians are more familiarised with the diagnosis of carious lesions in this manner and we must not forget the relative lack of experience of examiners using transillumination; the latter is a much newer method and is less common in the praxis of clinicians [12,14,20]. Notwithstanding this, the Kappa coefficient values indicate that there is reasonably good agreement, both at intra-examiner and at inter-examiner levels for each of the methods used but offering nonetheless better results for the case of transillumination [27]. These results are in line with those obtained by Peers et al. some years ago in their study [24].

Following from this, it is important to consider that prior to routinely applying transillumination in clinical praxis, better results are needed for the values of sensitivity, specificity, and AUC in order to substitute X-rays across the board. To attain this, clinicians need to become familiarised with its use as a diagnostic technique. The authors of in vitro studies coincide in that the results that they obtain also present a degree of limitation as they cannot be directly extrapolated to clinical praxis because the conditions in which they are functioning are those of a laboratory setting, i.e., ideal ones [11,12].

## 6. Conclusions

Following an analysis of the bibliography available and with the limitations set forth herein, we can conclude that transillumination is a method offering moderate validity in the diagnosis of carious lesions in dentine, given that we have measured a sensitivity value of 0.71, a specificity value of 0.86 and furthermore, AUC is measured at 0.76, not reaching hence the level of 80%. There is no strong evidence that may enable us to affirm that transillumination may fully substitute X-rays in the complementary diagnosis of carious lesions. Further studies are suggested to focus on the sensitivity and specificity of this method; this is necessary if a reliable conclusion is to be reached regarding its usefulness in daily diagnostic praxis in relation to early-stage caries on interproximal surfaces.

## Figures and Tables

**Table 1:** Results of the systematic analysis.

AUTHOR/Year	*n*	Sn	Sp	AUC	GS	VV/VT
Abogazalah N. 2017 [11]	85	TRIL: 0.68 Rxd: 0.50	TRIL: 0.93 Rxd: 0.64	TRIL: 0.81 Rxd: 0.61	T	VT
Abogazalah N. 2018 [15]	30	0.97	0.93	0.92	T	VT
Ástvaldsdóttir Á. 2012 [12]	112	TRIL D1: 0.7/D3: 0.57 Rxd D1: 0.37/D3: 0.47	TRIL D1: 0.87/D3: 0.93 Rxd D1: 0.91/D3: 0.97	TRIL D1: 0.82/D3: 0.88 Rxd D1: 0.64/D3: 0.84	H	VT
Baltacioglu IH. 2017 [6]	52			TRIL: 0.81 Rxd: 0.69	C	VV
Chawla N. 2012 [13]	135			TRIL D1: 0.66/D3: 0.81 Rxd D1:0.68/D3: 0.86	H	VT
Kühnisch J. 2016 [18]	127	TRIL: 0.291 Rxd: 0.961		TRIL: 0.65 Rxd: 0.984	C	VV
Lederer A. 2019a [16]	120	NILT D3: 0.82	NILT D3: 0.98	NILT D3: 0.90	T	VT
Lederer A. 2019b [17]	100	NILT D3: 0.55	NILT D3: 0.98		T	VT
Maia AMA. 2011 [14]	28	TRIL: 0.88 Rxd: 0.44	TRIL: 0.72 Rxd: 0.61		H	VT
Ozkan G. 2017 [7]	157	TRIL: 0.82 Rxd:0.83	TRIL:0.20 Rxd:0.60	TRIL:0.51 Rxd: 0.71	C	VV
Simon JC. 2017 [19]	30	TRIL: 0.53 Rxd: 0.23	TRIL: 0.86 Rxd: 0.96		H	VV

*n*: sample size; Sn: Sensitivity; Sp: Specificity; AUC: Area Under the ROC Curve; D1: caries lesion in enamel; D3: caries lesion in dentine; VV: in vivo studies; VT: in vitro studies; TRIL: transillumination, NILT: Near-Infrared Light Transillumination; Rxd: digital radiography; GS: gold standard; C: clinic diagnosis; H: hisological diagnosis; T: Tomography.

**Table 2:** QUADAS-2 qualitative analysis.

Study	Risk of Bias				Concerns Regarding Applicability		
	Patient Selection	Index Test	Reference Standard	Flow and Timing	Patient Selection	Index Test	Reference Standard
Abogazalah N. 2017 [11]	?	☺	☺	☺	☺	☺	☺
Abogazalah N. 2018 [15]	☺	☺	☺	☺	☺	☺	☺
Ástvaldsdóttir Á. 2012 [12]	☺	?	☺	☺	☺	☺	☺
Baltacioglu IH. 2017 [6]	☹	?	☺	☺	☺	☺	☺
Chawla N. 2012 [13]	☺	☺	☺	☺	☺	☺	☺
Kühnisch J. 2016 [18]	☹	☺	?	☺	☺	☺	☺
Lederer A. 2019a [16]	☺	☺	☺	☺	☺	☺	☺
Lederer A. 2019b [17]	☺	☺	☺	☺	☺	☺	☺
Maia AMA. 2011 [14]	?	☺	☺	?	☺	☺	☺
Ozkan G. 2017 [7]	☹	☺	?	☺	☹	☺	☺
Simon JC. 2017 [19]	?	☺	☺	☺	☺	☹	☺

☺ Low probability; ☹ High probability; ? Uncertain probability.

## Supplementary Materials

The following are available online at https://www.mdpi.com/2077-0383/9/2/420/s1, Figure S1: One study removed for sensitivity, Figure S2: One study removed for specificity, Figure S3: One study removed for AUC.
