# Supplementary material for: Diagnostic Validity of Digital Imaging Fiber-Optic Transillumination (DIFOTI) and Near-Infrared Light Transillumination (NILT) for Caries in Dentine
Source: J Clin Med. 2020 Feb 4;9(2):420. doi: 10.3390/jcm9020420 (PMC7073697; doi:10.3390/jcm9020420)
Supplement: Supplementary file 1 [file jcm-09-00420-s001.pdf]

Study name	Subgroup within study	Point	Lower limit	Upper limit	Z-Value	p-Value
		Statistics with study removed				
Abogazalah 2017	In Vitro	0.695	0.522	0.827	2.196	0.028
Abogazalah 2018	In Vitro	0.656	0.500	0.784	1.962	0.050
Ástvaldsdóttir 2012	In Vitro	0.711	0.535	0.840	2.319	0.020
Kühnisch 2016	In Vivo	0.727	0.613	0.818	3.679	<0.001
Lederer 2019a	In Vitro	0.667	0.506	0.797	2.031	0.042
Lederer 2019b	In Vitro	0.712	0.539	0.840	2.372	0.018
Maia 2011	In Vitro	0.663	0.503	0.793	1.992	0.046
Ozkan 2017	In Vivo	0.666	0.507	0.793	2.049	0.040
Simon 2017	In Vivo	0.710	0.548	0.832	2.494	0.013
		**0.690**	**0.539**	**0.809**	**2.441**	**0.015**

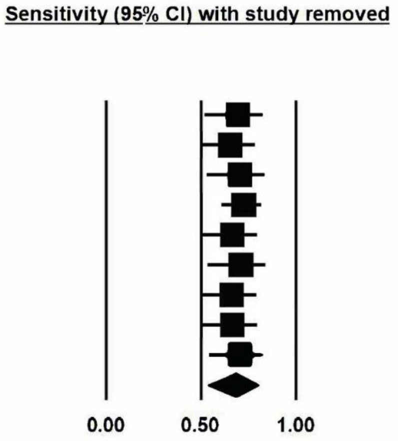

Figure S1. One study removed for sensitivity.

Study name	Subgroup within study	Point	Lower limit	Upper limit	Z-Value	p-Value
		Statistics with study removed				
Abogazalah 2017	In Vitro	0.881	0.554	0.978	2.197	0.028
Abogazalah 2018	In Vitro	0.882	0.563	0.977	2.245	0.025
Ástvaldsdóttir 2012	In Vitro	0.881	0.552	0.978	2.187	0.029
Lederer 2019a	In Vitro	0.861	0.535	0.971	2.122	0.034
Lederer 2019b	In Vitro	0.862	0.534	0.971	2.117	0.034
Maia 2011	In Vitro	0.905	0.586	0.984	2.319	0.020
Ozkan 2017	In Vivo	0.926	0.855	0.964	6.577	<0.001
Simon 2017	In Vivo	0.893	0.575	0.981	2.286	0.022
		**0.889**	**0.612**	**0.976**	**2.509**	**0.012**

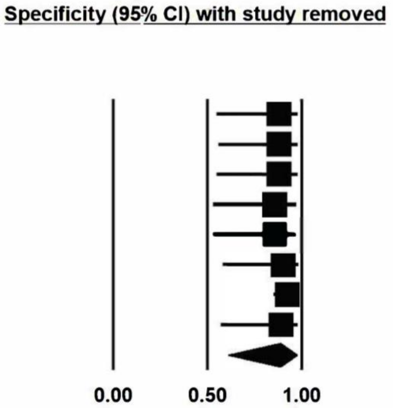

Figure S2. One study removed for specificity.

Study name	Subgroup within study	Point	Lower limit	Upper limit	Z-Value	p-Value
		Statistics with study removed				
Abogazalah 2017	In Vitro	0.784	0.652	0.876	3.820	<0.001
Abogazalah 2018	In Vitro	0.769	0.644	0.859	3.866	<0.001
Ástvaldsdóttir 2012	In Vitro	0.769	0.643	0.860	3.852	<0.001
Baltacioglu 2017	In Vivo	0.800	0.672	0.886	4.056	<0.001
Chawla 2012	In Vitro	0.784	0.649	0.877	3.751	<0.001
Kühnisch 2016	In Vivo	0.806	0.672	0.894	3.945	<0.001
Lederer 2019a	In Vitro	0.763	0.641	0.853	3.890	<0.001
Ozkan 2017	In Vivo	0.815	0.728	0.879	5.843	<0.001
		**0.787**	**0.671**	**0.870**	**4.323**	**<0.001**

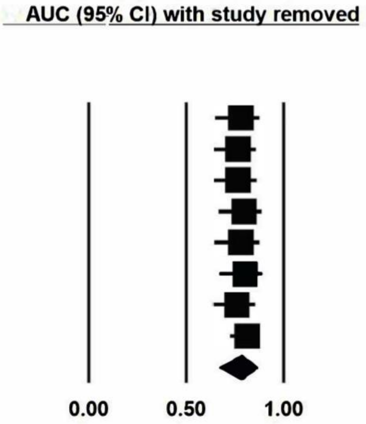

Figure S3. One study removed for AUC.
